# Draft Genome Sequences of Phytase Active Endophytic and Epiphytic Erwinia gerundensis Isolated from Wheat (Triticum aestivum) Seeds

**DOI:** 10.1128/mra.01073-22

**Published:** 2022-12-07

**Authors:** Jonathan Sølve, Tue Kjærgaard Nielsen, Mette Haubjerg Nicolaisen, Frederik Bak

**Affiliations:** a University of Copenhagen, Department of Plant and Environmental Sciences, Copenhagen, Denmark; University of Arizona

## Abstract

Here, we provide draft genome sequences of an epiphytic strain (HEP01) and an endophytic strain (HEN01) of Erwinia gerundensis, isolated from wheat (Triticum aestivum) seeds. Genome sizes of HEP01 and HEN01 were 3,771,322 bp and 3,750,048 bp, respectively. HEP01 and HEN01 carried one plasmid each with sizes of 565,617 bp and 576,781 bp, respectively. Both showed phenotypic phytase activity.

## ANNOUNCEMENT

Species of the genus *Erwinia* are mostly plant related and many have been described as plant pathogens (1–3). Erwinia gerundensis was recently discovered and described as a facultative anaerobic nonpathogenic plant-associated species (4). It has been isolated from different pome fruit trees, including apple and pear; peach trees; perennial ryegrass seeds; and almond tree leaves (4–6). The genome sequences of two *E. gerundensis* strains isolated from wheat seeds provided here can contribute to a better understanding of the ecological role of this species during seed germination, specifically their involvement in phytate degradation.

We isolated two distinct strains of *E. gerundensis* from wheat seeds (cv. Heerup; Sejet Plant Breeding, Horsens, Denmark), namely, one from the surface (epiphyte) and the other from crushed surface-sterilized seeds (endophyte).

Surface sterilization was achieved by submerging seeds for 2 min (gentle shaking by hand) in 2% hypochlorite solution and then washing seeds in sterile water once, in 70% ethanol for 5 min, and in sterile water three times. Surface-sterilized seeds were crushed, dissolved, and diluted in saline water (0.9% NaCl) under sterile conditions prior to sonication (2 times for 2 min) and vortexing (30 sec at maximum speed). Epiphytic isolation was carried out by vortexing nonsterile seeds in saline water (30 sec at maximum speed). The liquid phase was spread plated onto tryptic soy agar (TSA) and incubated at 20°C for 72 h until pale-yellow colonies appeared. A single colony was picked from each isolation setup and purified by restreaking on TSA plates.

Phosphorous (P) in wheat seeds is stored primarily as phytate (myo-inositol hexakisphosphate) and can be enzymatically broken down by phytases releasing inorganic P (7, 8). Extracellular phytase activity was observed for both HEP01 and HEN01 at pH 6.0 to 7.0 using the qualitative phytase screening medium based on reference 9, with the addition of the bromophenol blue as a pH indicator.

Bacterial genomic DNA for Nanopore sequencing was extracted from overnight cultures grown in tryptic soy broth at 20°C using the Gentra Puregene yeast/bact kit B (Qiagen Sciences, Germantown, MD). Library preparation with multiplexing was performed using the rapid barcoding kit 96 SQK-RBK101.96 (Oxford Nanopore Technologies, Oxford, UK). Whole-genome sequencing was performed on a MinION instrument (Oxford Nanopore Technologies), using a R9.4.1 flow cell. No size selection was performed prior to library preparation.

The obtained reads were base called and demultiplexed in superaccurate mode using Guppy v6.0.1 with the dna_r9.4.1_450bps_sup.cfg model. Default parameters were used for all software. The adapters were trimmed using Porechop v0.2.4 (https://github.com/rrwick/Porechop) prior to genome assembly using Flye v2.9 (10). The assembled genomes were polished twice with Medaka v1.1.1 (https://github.com/nanoporetech/medaka). Prokka v1.14.6 (11) was used for genome annotation. Genome completeness and redundancy were estimated using checkM v1.1.6 (12). The 16S rRNA gene sequences from HEN01 and HEP01 were blasted against the EzBioCloud (13) 16S rRNA database where both strains showed a 99.48% similarity with Erwinia gerundensis EM595. The genome sequences of the two strains were compared by calculating the average nucleotide identity (ANI) values using EzBioCloud (14). Genomic features are included in Table 1.

**TABLE 1:** Overview of sequencing and genome features of the two *E. gerundensis* strains isolated from wheat seeds

Parameter	Data by *E. gerundensis* strain	
	HEN01 (endophyte)	HEP01 (epiphyte)
Total no. of reads	114,862	91,519
Total read length (bp)	832,247,939	519,192,820
*N*_50_ value (bp)	13,734	12,595
Mean coverage (×)	166	69
Genome size (mbp)	3,750,048	3,771,322
Plasmid size (bp)	576,781	565,617
GC content (%)	55.38	55.38
Genome completeness (%)	100	100
Genome contamination (%)	0.25	0.25
No. of coding sequences	3,879	3,927
No. of rRNAs	22	22
No. of tRNAs	79	79
ANI value (%)	98.85	98.85
Genome accession no.	JAOSHP000000000	JAOSHQ000000000
SRA accession no.	SRX17792849	SRX17792850

HEP01 and HEN01 contained one gene annotated as a hypothetical protein that had 100% protein identity with an inositol phosphatase from Erwinia gerundensis identified using DIAMOND blastx (15) on a database with curated phytase gene sequences from UniProt (16).

### Data availability.

The draft genome sequences reported here were deposited in GenBank under the BioProject accession number PRJNA885388. The raw sequence reads (adapters removed) and genome assembly accession numbers are listed in Table 1.
